# Ancient Mitochondrial Genomes Reveal Extensive Genetic Influence of the Steppe Pastoralists in Western Xinjiang

**DOI:** 10.3389/fgene.2021.740167

**Published:** 2021-09-22

**Authors:** Chao Ning, Hong-Xiang Zheng, Fan Zhang, Sihao Wu, Chunxiang Li, Yongbin Zhao, Yang Xu, Dong Wei, Yong Wu, Shizhu Gao, Li Jin, Yinqiu Cui

**Affiliations:** ^1^School of Life Sciences, Jilin University, Changchun, China; ^2^Max Planck Institute for the Science of Human History, Jena, Germany; ^3^State Key Laboratory of Genetic Engineering, School of Life Sciences, and Human Phenome Institute, Fudan University, Shanghai, China; ^4^College of Life Science, Jilin Normal University, Siping, China; ^5^School of Archaeology, Jilin University, Changchun, China; ^6^Xinjiang Cultural Relics and Archaeology Institute, Urumchi, China; ^7^School of Pharmaceutical Sciences, Jilin University, Changchun, China

**Keywords:** mitochondrial genome, ancient DNA, Eurasian Steppe, Silk Road, Andronovo

## Abstract

The population prehistory of Xinjiang has been a hot topic among geneticists, linguists, and archaeologists. Current ancient DNA studies in Xinjiang exclusively suggest an admixture model for the populations in Xinjiang since the early Bronze Age. However, almost all of these studies focused on the northern and eastern parts of Xinjiang; the prehistoric demographic processes that occurred in western Xinjiang have been seldomly reported. By analyzing complete mitochondrial sequences from the Xiabandi (XBD) cemetery (3,500–3,300 BP), the up-to-date earliest cemetery excavated in western Xinjiang, we show that all the XBD mitochondrial sequences fall within two different West Eurasian mitochondrial DNA (mtDNA) pools, indicating that the migrants into western Xinjiang from west Eurasians were a consequence of the early expansion of the middle and late Bronze Age steppe pastoralists (Steppe_MLBA), admixed with the indigenous populations from Central Asia. Our study provides genetic links for an early existence of the Indo-Iranian language in southwestern Xinjiang and suggests that the existence of Andronovo culture in western Xinjiang involved not only the dispersal of ideas but also population movement.

## Introduction

Recent archaeogenetic studies showed that the expansion of western steppe herders (WSHs) had a marked impact on the demographic, cultural, social and linguistic development since the third millennium BCE on the Eurasian continent (Allentoft et al., 2015; Haak et al., 2015; Damgaard et al., 2018a; Jeong et al., 2018, 2020; Narasimhan et al., 2019; Wang C. C. et al., 2021). One of the earliest representatives, known as the Yamnaya culture (ca. 3,300–2,700 BCE) from the Pontic–Caspian steppe migrated into Europe and Asia, bringing with them metallurgy, animal herding skills, and possibly the Indo-European languages (Frachetti, 2009; Allentoft et al., 2015; Haak et al., 2015). By the middle and late Bronze Age, the Sintashta culture (ca. 2,200–1,800 BCE) arose near the Urals and succeeded a majority of ancestry from the preceding Yamnaya culture. It carried a similar genetic profile with the Srubnaya and the Andronovo cultures that spread over a large part of the Eurasia landmass, extending westward into Europe, southward into Central Asia and the India subcontinent, and eastward into the Mongolian Plateau (Allentoft et al., 2015; Haak et al., 2015; Damgaard et al., 2018b; Narasimhan et al., 2019; Jeong et al., 2020; Wang C. C. et al., 2021). A number of studies provided the evidence that the steppe cultures from western Eurasia had also integrated into the early Bronze Age cultures of western China. A recent archaeobotanical study showed that both wheat and barley had already spread to the Altai Mountains as early as 5,200 years ago (Zhou et al., 2020). Additionally, domesticated sheep and cattle were also observed in the prehistoric cultures of northwestern China (e.g., Majiayao culture, 3,550–2,850 BC; Qijia, 2,450–1,650 BC) (Fu et al., 2009). The cultural influences from WSHs suggested that ancient mobile pastoralists had played an extremely significant role in the prehistoric *trans-*Eurasian exchanges and the formation of agropastoralism.

Located at the intersection of the ancient “Silk Road,” Xinjiang has played an important role in bridging the exchanges of cultures, goods, languages, and population movements (Wood, 2002). A recent genome-wide study on 951 Uyghurs in Xinjiang revealed a complex demographic history of the present-day populations in this region. Four major ancestral components were identified, namely, European, South Asian, Siberian, and East Asian (Feng et al., 2017). Two waves of admixtures were further characterized, with the first wave dating back as early as 3,750 years ago (Feng et al., 2017). However, human populations always underwent frequent population migrations, admixtures, and replacements, which made it difficult to reflect the true ancestral components and population dynamics using extant population data alone. The high level of genetic diversity of present-day Xinjiang people was likely a result of recent admixture events. The opening of the “Silk Road” made the exchanges of different populations in Xinjiang more frequent than ever. In contrast, ancient DNA study has been proven to be a powerful tool to reconstruct human prehistory by providing direct tests on samples from a certain period. Previous genetic studies have delineated that modern and ancient Xinjiang populations had maternal genetic affinities with both the eastern and western Eurasians, displaying high genetic diversity and admixture (Yao et al., 2004; Li et al., 2010; Zhang et al., 2010; Zheng et al., 2017; Wang W. et al., 2021). A recent paleogenomic study on the Iron Age Shirenzigou individuals from the eastern Tianshan mountains further confirmed the previous observations and characterized that the West Eurasian ancestry was likely to be related to the Early Bronze Age steppe pastoralists such as Yamnaya and/or Afanasievo than the chronologically more recent Sintashta and Andronovo cultures (Ning et al., 2019). Wang W. et al. (2021) retrieved the whole mitochondrial genomes of ancient Xinjiang populations from the Bronze Age to Historic Era. Their results revealed that the Bronze Age Xinjiang populations had genetic affinities with Steppe-related and Northeastern Asian populations (Wang W. et al., 2021). All of the above studies had proven the very complex demographic landscape of the ancient Xinjiang populations.

However, all those ancient DNA studies of Xinjiang were confined to the northern and eastern parts of this region. Considering the large geographic range and diverse ecosystems of Xinjiang, such studies in western Xinjiang are in great need to gain a more comprehensive understanding of the prehistoric demography of Xinjiang populations. In recent decades, a number of cultural remains and archaeological sites in western Xinjiang, showing the traits that are characteristic of the middle and late Bronze Age Eurasian Steppe (Steppe_MLBA) cultures (e.g., Sintashta and Andronovo) (Shao and Zhang, 2019), were investigated. However, the stable isotope analysis of the Bronze Age Xiabandi (XBD) population provided direct evidence of wheat and millet consumption in the eastern part of the Pamir Plateau (Zhang et al., 2016), suggesting that the possible East–West cultural interactions and communications in westernmost Xinjiang can be dated to 1,500 BC. A craniometry study on individuals from the Liushui cemetery (∼2,950 BP) in western Xinjiang also showed that the population was already admixed between the East and West Eurasians but with the majority inherited from the former (Zhang et al., 2011). The above research presented a complex and confusing scenario of western Xinjiang. More genetic studies on ancient populations in this region will undoubtedly provide important clues to the issue.

In this study, we collected 15 ancient samples from the XBD cemetery, the earliest archaeological site excavated in western Xinjiang to the best of our knowledge. We then enriched and sequenced the complete mitochondrial genomes of the XBD individuals through designed target probes. By comparing the mitochondrial DNA (mtDNA) of the XBD individuals with that of ancient and extant Eurasians, we explored the early population movement in western Xinjiang.

## Materials and Methods

### Archaeological Background, Sampling, and Sequencing

**FIGURE 1:**
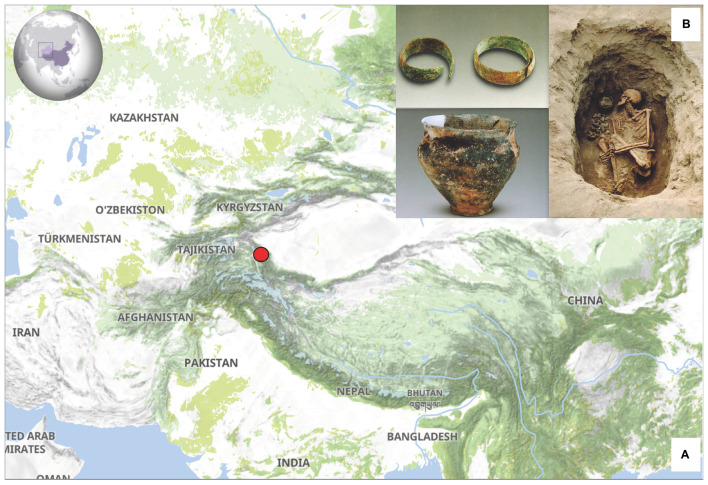
**(A)** Geographic location and **(B)** representative archaeological excavations of the Xiabandi (XBD) cemetery.

The XBD cemetery is located in the westernmost region of Xinjiang, adjacent to the eastern edge of the Pamir Plateau (Figure 1A). This region lies at the intersection of the southern and northern branches of the historical “Silk Road,” making it an important melting place for populations from East and Central Asia, as well as those from the Eurasia steppe. The XBD cemetery was investigated by Xinjiang Cultural Relics and Archaeology Institute in 2003 and 2004. The whole cemetery can be divided into three phases, the earliest of which was dated to the Bronze Age (3,500–3,300 BP), and the remaining two phages were dated to Han-Tang (∼2,200–1,300 BP) and Ming-Qing dynasties (∼600–300 BP) (Wu, 2012). The excavations of the jars with contracting neck, the bowls, the trumpet-shaped earrings, as well as the wide band-shaped bracelets in the first phase suggest that the XBD cemetery belongs to the Andronovo culture (Figure 1B; Wu, 2012). The cemetery contained 92 burials from the Bronze Age, but only 27 human skeletons were excavated. We selected 15 well-preserved skulls and sampled the intact and sound teeth for genetic research (Table 1; Supplementary Table 1A). The permission for the use of the 15 Bronze Age samples of the XBD cemetery was obtained from Xinjiang Cultural Relics and Archaeology Institute.

**TABLE 1:** Summary of XBD individuals included in this study.

Sample ID	Biological sex	Archaeological dating (BP)	Archaeological culture	Mean coverage	Contamination (%)	Haplogroup
XBD-M18	Female	3,500–3,300	Andronovo	1,578	1.3	R1b
XBD-40	Male	3,500–3,300	Andronovo	1,622	5.8	H6a1a
XBD-M24	Male	3,500–3,300	Andronovo	1,143	1.6	H5b
XBD-M29	Male	3,500–3,300	Andronovo	4,130	0.6	U4a1
XBD-M10	Male	3,500–3,300	Andronovo	187	2.8	U4c1
XBD-M9	Female	3,500–3,300	Andronovo	3,410	3.4	H6a1a
XBD-M36	Female	3,500–3,300	Andronovo	1,881	1.9	U1a1c1
XBD-36	Female	3,500–3,300	Andronovo	2,023	1.4	R1b1
XBD-M45	Male	3,500–3,300	Andronovo	897	2.6	H11b
XBD-M46	Female	3,500–3,300	Andronovo	383	2	HV14
XBD-37	Female	3,500–3,300	Andronovo	1,308	4.8	U2e2a1d
XBD-M48	Male	3,500–3,300	Andronovo	576	2.1	U2e3
XBD-M17	Male	3,500–3,300	Andronovo	812	2.5	I4a
XBD-M14	Male	3,500–3,300	Andronovo	1,084	4.1	U2e1
XBD-38	Male	3,500–3,300	Andronovo	581	1.1	T2a1b1

*XBD, Xiabandi.*

DNA was extracted from teeth powder (∼50 mg) with the method previously described (Ning et al., 2016). The libraries were prepared with the NEBNext Ultra DNA Library preparation kit (New England Biolabs, United Kingdom) following the manufacturer’s protocol but with a 1:20 dilution of the adapter during the ligation step. The quality and concentration of the libraries were determined on an Agilent Bioanalyzer 2100 (Agilent Technologies, Palo Alto, CA, United States). Subsequently, targeted enrichment of the mtDNA was conducted with the MitoCap^TM^ kit (MyGenostics, Beijing, China). Sequencing was carried out on an Illumina HiSeq 2000 platform at Novogene Inc. (Beijing, China).

### Sequence Mapping and Mitochondrial DNA Haplogroup Determination

Raw data was processed using EAGER v1.92.50 with default parameters, a pipeline specially designed to deal with ancient DNA data (Peltzer et al., 2016). Quality assessment was performed with FastQC software (Andrews, 2010). The adapters were trimmed with AdapterRemoval v2.2.0 with a minimum overlap of 1 bp and base quality larger than 20 (Schubert et al., 2016). Reads shorter than 30 bp were disregarded. BWA v0.7.12 was used to align the reads to the Revised Cambridge Reference Sequences (rCRS) with seed disabled (*-l 2000*). The duplicate reads were removed by the DeDup v0.12.1 (Peltzer et al., 2016). Ancient DNA deamination rates were calculated with MapDamage v2.0 (Jonsson et al., 2013). Single-nucleotide polymorphisms (SNPs) and insertions and deletions (INDELs) were called using SNVer-0.5.2 (Wei et al., 2011) and were checked by visual inspection. We used trimmed 10 bp at both 3′ and 5′ ends with TrimBam function in the BamUtils v1.0.13^1^ to minimize the bias caused by ancient DNA deamination. The mitochondrial haplogroups were determined with Haplogrep2 (Weissensteiner et al., 2016) according to PhyloTree build 17 (Van Oven, 2015).

### Analysis of Xiabandi Mitochondrial DNA Genomes

Haplogroup frequencies were estimated by simple counting. A principal component analysis (PCA) based on the frequencies of sub-haplogroups was performed with the R libraries “factoextra”, “FactoMineR”, and “ggplot2”.

The coalescence time of each lineage was estimated using the ρ statistic-based method and the maximum likelihood (ML) method implemented in PAML software v4.9g (Yang, 2007) with the Soares rate for complete mitochondrial genomes (Soares et al., 2009). For the ρ-based method, the corresponding standard deviation (SD) was calculated following published methods (Saillard et al., 2000). With the knowledge of coalescence time of each haplogroup estimated by contemporary samples, a Bayesian method implemented in BEAST software v1.8.0 was used to infer the time of XBD samples (Drummond and Rambaut, 2007).

## Results

### Mitochondrial DNA Authentication and Contamination Assessment

Strict contamination precautions for ancient DNA were taken, and wet lab works were carried out in a dedicated clean room facility specially designed for ancient DNA studies at Jilin University. All samples showed a short fragment length (55–90 base pairs) and postmortem chemical modifications at 3′ and 5′ ends that are expected for ancient DNA (Dabney et al., 2013). The contamination rates of those samples were further quantitatively evaluated with contamMix v1.0-10 (Fu et al., 2013). As a result, we obtained a low level of modern human DNA contaminations (<5%) with the exception of one individual to be 5.8% (Table 1), which restricted our analysis to the deaminated reads to rule out modern human contamination. By applying the above criteria, we confirmed the authentication of our ancient data.

### Major Bronze Age Steppe Pastoralist Origin of the Xiabandi Mitochondrial Haplogroups

**FIGURE 2:**
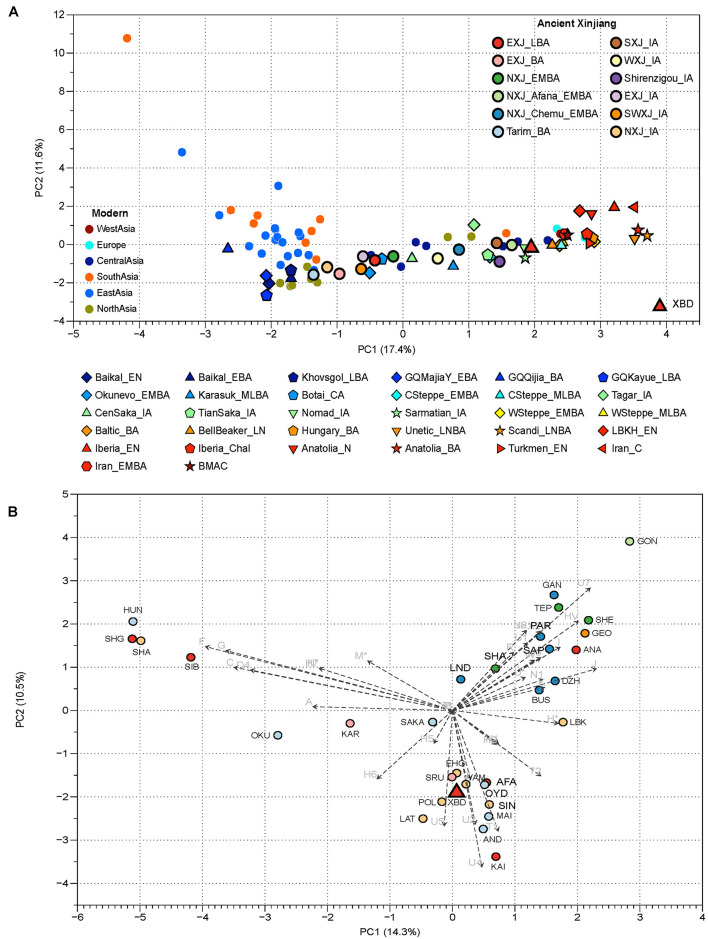
Principal component analysis (PCA) based on mitochondrial DNA (mtDNA) haplogroup frequencies of ancient and present-day Eurasian populations. **(A)** The PCA constructed by populations from Xinjiang and neighboring regions. The different combinations of color and scheme on the plot represent different groups, present-day populations are marked with a circle; circles with dark black frames represent the published ancient Xinjiang samples, while shapes with light black frames are published ancient populations from the Eurasian continent. The single red triangle is the XBD populations in our study. NXJ, northern Xinjiang; EXJ, eastern Xinjiang; SXJ, southern Xinjiang; SWXJ, southwestern Xinjiang; WXJ, western Xinjiang. Detailed descriptions and references of comparative populations are provided in Supplementary Tables 1B, C. **(B)** The PCA constructed on ancient populations from the Eurasian continent. The two dimensions account for 25.8% of the total variance. Haplogroup contributions are represented by loading vectors marked as grep arrows. Population abbreviations are as follows: XBD, Xiabandi; SMK, Shamanka_EBA; ESHG, Scandinavian hunter-gatherers; ANA, Anatolia_N; LAT, Mesolithic/Neolithic Hunter gatherers from Lativ; LBK, LBK_EN; SIB, ancient Siberians; AFA, Afanasievo; KAI, Kairan_MLBA; AND, Andronovo; HUN, Tianshan_Hun; SAK, Tianshan_Saka; MAI, Maitan_MLBA; OKU, Okunevo; OYD, Oy_Dzhaylau_MLBA; POL, Poltavka; SIN, Sintashta; SRU, Srubnaya; YAM, Yamnaya; KAR, Karasuk; BUS, Bustan_BA; DZH, Dzharkutan_BA; SAP, Sappali_Tepe_BA; GEO, Geoksyur_EN; GAN, Ganj_Dareh; IND, Indus_Periphery_BA; PAR, Parkhai_EN; SHA, Shahr_I_Sokhta_BA; SEH, Seh_Gabi; TEP, Tepe_Hissar_CHL/EN; SHE, She_Gabi_ChL/EN. Detailed descriptions and references of comparative populations are provided in Supplementary Table 1D.

We obtained complete mitochondrial sequences to an average coverage of 187- to 4,130-fold across all 15 individuals sequenced in this study. A total of 14 different mitochondrial haplogroups belonging to five macro-haplogroups, such as U, H, T, R, and I, were observed (Table 1; Supplementary Table 1A). All these haplogroups commonly appear in present-day populations from Europe, Central Asia, and Central/Western steppe, and are uniformly considered to be of West Eurasian origin. Two out of the 14 haplogroups, namely, HV14 and U1a1c1, are prevalent in the extant Central and Western Asians (Palanichamy et al., 2015; Narasimhan et al., 2019; Shamoon-Pour et al., 2019). Haplogroup HV14 was present in two ancient individuals from Central Asia, one (3,000–2,200 BCE) from Turkmenistan and the other (2,100–1,800 BCE) from Uzbekistan (Narasimhan et al., 2019). Similarly, U1a1c1 was found in a historical individual (680–649 CE) from the Pontic steppe (Narasimhan et al., 2019) and the Bronze Age individuals from Iran (3,328–3,022 BCE) and Turkmenistan (2,500–1,700 BCE), who were associated with the bactria-margiana archaeological complex (BMAC). These results suggest that haplogroup HV14 and U1a1c1 are probably Central or Western Asian origin. The remaining 12 haplogroups (I4a, H6a1a, H5b, H11b, R1b, R1b1, T2a1b1, U2e1, U2e2a1d, U2e3, U4a1, U4c1), however, were detected in the Bronze Age steppe pastoralists, the ancient Xinjiang groups, and the prehistoric populations in Europe. For example, haplogroup U2e, the most abundant type in XBD (20%), was found in high frequency in the Sintashta (11.6%) and Andronovo (14.3%) populations. Haplogroup U4a1, which had a high frequency in the Andronovo population (19%), was also observed in one individual associated with the Afanasievo culture (Allentoft et al., 2015). Beyond their wide distributions in the Bronze Age steppe pastoralists, several haplogroups were detected in some pre-Bronze Age hunter-gatherers from the central steppe as well. For example, haplogroup R1b was identified in an Upper Paleolithic individual from the left bank of the Yenisei River dated to around 14,000 BP. In addition, haplogroups U4a1, R1b1, and U2e3 were observed in the Botai culture from northern Kazakhstan and in Eastern Europe hunter-gatherer (Mathieson et al., 2015; Fu et al., 2016; Mittnik et al., 2018). Notably, haplogroups I4a, R1b1, and U2e2a1d were found in individuals who were associated with the BMAC culture and dated to the beginning of this culture 451 in Central Asia. These earlier individuals shared the substratum with the BMAC group but harbored additional Bronze Age steppe pastoralist ancestry than the main BMAC group as evidenced by the autosomal data (Narasimhan et al., 2019). Genetic frequency-based principal component analysis (PCA) agrees with what we have observed in the mitochondrial haplogroup distributions that the XBD falls within the western Eurasian cluster (right) formed by the ancient nomads, WSteppe_EMBA and WSteppe_MLBA, represented by the Yamnaya and Andronovo, respectively (Figure 2A; Supplementary Table 1F). When compared to the other ancient populations from Xinjiang, we found that the XBD clustered with the NXJ_Afana_EMBA and Shirenzigou_IA, both of which were previously proven to share significant genetic affinity with the Bronze Age steppe pastoralists (Ning et al., 2019; Wang W. et al., 2021). In a finer scale PCA plot, the XBD also clustered with multiple WSH groups but shifted toward the South/Central Asian populations (top right) slightly (Figure 2B; Supplementary Table 1E), documenting that the majority of XBD mitochondrial haplogroups (12/14) can trace their origin from the Eurasia steppe pastoralist while the minority (2/14) from West or Central Asia.

### Expansion of the Bronze Age Steppe Pastoralists as a Dynamic Process to Form the Genetic Landscape of Xiabandi Individuals

**FIGURE 3:**
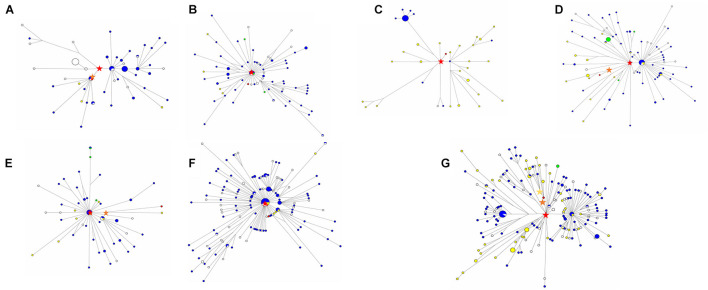
Median-joining networks of star-like Xiabandi (XBD)-related mitochondrial DNA (mtDNA) haplogroups. The median-joining network of haplogroup U2e2a1d **(A)**, I4a **(B)**, U1a1c1 **(C)**, U4a1 **(D)**, U4c1 **(E)**, H6a1a **(F)**, and U2e1 **(G)** constructed from the complete mitochondrial sequences of both XBD and relevant present-day Eurasians. The red stars mark XBD individuals, the blue plots represent the Europeans, the green plots represent North Asians, the yellow plots represent West and East Asians, and the white plots represent the individuals that are of unknown location. The red stars are the original haplogroup, the orange star in **(A)** is haplogroup U2e2a1d, the orange star in **(E)** is haplogroup U4c1 + A827G, the orange star in **(D)** is haplogroup U4a1 + C7868T + A13773G, the orange star in **(G)** is haplogroup U2e1 + C2526T + G12618A, and the yellow star in **(G)** is haplogroup U2e1e.

We used 540 present-day mitochondrial sequences obtained from PhyloTree database (van Oven and Kayser, 2009) who were genetically close to XBD individuals to construct the mtDNA phylogeny (Supplementary Table 2). Coalescence times of 14 mtDNA haplogroups related to XBD samples were estimated employing the ρ-based and the ML methods. The estimates obtained by both methods showed consistency (Table 2), suggesting the reliability of our estimates. Out of the 14 haplogroups, seven (U2e2a1d, I4a, U1a1c1, U4a1, U4c1, H6a1a, and U2e1) showed a star-like phylogeny of their ancestral node, indicating strong population expansions. Among the seven star-like lineages (Figure 3), four (I4a, H6a1a, U2e2a1d, and U4c1) were estimated of rather time to most recent common ancestor (TMRCA) of <6,000 BP with the most recent expansion lineage (U2e2a1d) estimated at approximately 4,470 BP (Table 2). This time is within the range of the presence of Early Bronze Age steppe pastoralists represented by the Yamnaya culture (3,300–2,700 BCE) in the Pontic steppe and the Afanasievo culture (3,300–2,500 BCE) in the Altai Mountains and fits well with the onset of the Sintashta culture (2,200–1,800 BCE). The Sintashta culture first emerged in the Urals at around 2,200 BC with multiple technological innovations, such as the earliest known chariots and training horses (Kristiansen and Larsson, 2005), and gave rise to the Andronovo culture (1,500–1,700 BC) (Kuznetsov, 2006; Hanks et al., 2007; Allentoft et al., 2015). Those innovations together with the populations quickly spread across much of the Eurasia Steppe (Narasimhan et al., 2019; Jeong et al., 2020). The genetic observations here, as well as the archaeological evidences, suggest that the XBD population originated in a large extent from the middle and late Bronze Age steppe pastoralists, who expanded to the western Xinjiang carrying their technologies along. However, two haplogroups (HV14 and U1a1c1) with Western or Central Asian origin were estimated of rather ancient TMRCA (14,660 and 11,290 BP, respectively) (Table 2), suggesting Western or Central Asian to be the source of these two haplogroups. A scenario to explain this phenomenon is that the Bronze Age steppe pastoralists expanded from the western and central steppe southward into Central Asia and admixed in a small scale with the indigenous populations there to form the ancestor of the XBD population, who then migrated eastward over the Pamir Plateau into western Xinjiang. This scenario is consistent with the recent ancient genomic study that the Bronze Age steppe pastoralists only marginally admixed with the indigenous population in Central Asia they met and moved farther southward into South Asia and admixed extensively with the local populations there (Narasimhan et al., 2019).

**TABLE 2:** TMRCA of XBD mtDNA haplogroups estimated from modern mtDNA genomes.

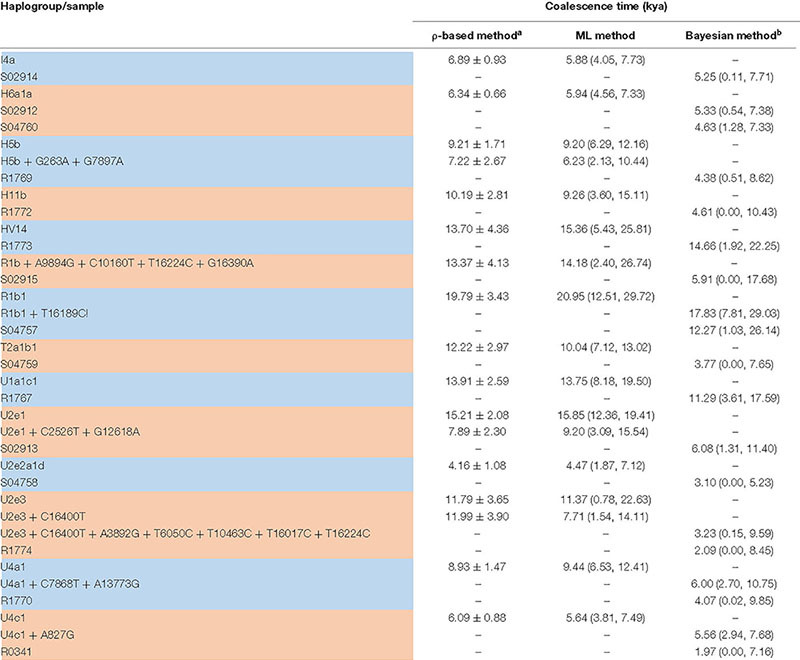

*^a^ We excluded Xiabandi and other ancient samples when using ρ-based method and ML method to estimate the coalescence time of each haplogroup.*

*^b^ We used a Bayesian method to infer the time of Xiabandi samples with the knowledge of coalescence time of each haplogroup estimated by contemporary samples.*

*ML, maximum likelihood; mtDNA, mitochondrial DNA; XBD, Xiabandi.*

## Discussion

The prehistory of Xinjiang is of considerable interest given its special geographic location in connecting the East and the West Eurasians. Multiple genetic studies showed that since the Bronze Age, the populations in Xinjiang had exhibited high genetic diversity and extensive admixture with various populations (Yao et al., 2004; Zhang et al., 2010). The admixture dating analysis based on linkage disequilibrium for the present-day populations in Xinjiang suggested multiple waves of admixture events (Zhang et al., 2010; Shan et al., 2014a,b; Feng et al., 2017). However, tracing the population prehistory with present-day individuals is prone to be distorted by recent admixture events, which is especially the case for Xinjiang populations because the opening of the well-known “Silk Road” made the gene flow among different populations in this region even more frequent. Ancient DNA studies in this region had shown that populations in eastern Xinjiang were already admixed between the East and the West Eurasians as early as the Second Millennium BCE (Li et al., 2010; Wang W. et al., 2021). Population genetic history of western Xinjiang, however, is still largely unknown. By analyzing the XBD mitochondrial genomes, we show here that XBD was genetically admixed from the middle and late Bronze Age steppe pastoralists and the indigenous populations in Central Asia, who probably migrated into Xinjiang through the Pamir Plateau.

The discovery of the Tocharian manuscript from the northern rim of the Tarim Basin and the Indo-Iranian manuscripts from the southern edge provides direct evidence for the dispersal of Indo-European languages into the region (Di Cosmo, 2002). It is now a general consensus among the linguistics that the dispersal of both languages is related to the Bronze Age Steppe herders (Walter, 1998). The Tocharians may have moved eastward earlier than the Indo-Iranians. The Tocharians are likely to be closely associated with the Afanasievo culture in the Altai Mountains who were a successor of the Yamnaya culture in the Pontic–Caspian Steppe. The middle and late Bronze Age steppe pastoralists, such as the Sintashta, Andronovo, and Srubnaya, are believed to be associated with the dispersal of Indo-Iranian languages (Lamberg-Karlovsky, 2002). The Iron Age individuals from northeastern Xinjiang were proved by autosomal DNA to harbor the Yamnaya/Afanasievo ancestry instead of the Steppe_MLBA, providing a strong genetic link of the “steppe hypothesis” over the “oasis hypothesis” and genetic support for the introduction of the Tocharian languages into Xinjiang (Ning et al., 2019). Our study here suggests a different genetic profile of totally west Eurasian origin, and provides a genetic link for the existence of Indo-Iranian languages in western Xinjiang at least 3,300 years ago.

## Conclusion

Taken together, the systematic mtDNA analysis on ancient samples from the westernmost part of Xinjiang provides us a unique opportunity to investigate the population origin of Xinjiang with a broader geography. We find that the 15 XBD individuals fall within the range of the ancient western Eurasian variation, and the formation of the ancestry legacy of XBD is related to the expansion of the middle and late Bronze Age steppe herders who might speak Indo-Iranian languages and admixed with the indigenous populations in the West or Central Asia during their expansion. Additionally, integrating the archaeological and genetic evidences in this study, the existence of the Andronovo culture in western Xinjiang involved not only the dispersal of ideas but also population movement. We recognize that such study on samples from a broader region and time sequences is required to obtain a more comprehensive understanding of the population prehistory of Xinjiang.

## Data Availability Statement

The datasets presented in this study can be found in online repositories. The names of the repository/repositories and accession number(s) can be found below: The BIG Data Center Genome Sequence Archive (GSA) under accession number HRA001154 (http://bigd.big.ac.cn/gsa-human).

## Author Contributions

YC, LJ, and SG conceived and supervised the study. CN, YZ, YX, and CL performed the laboratory work. YW and DW provided archaeological materials and associated information. CN, H-XZ, FZ, and SW analyzed the data. CN, YC, FZ, SG, and H-XZ wrote the manuscript with the input from all co-authors.

## Conflict of Interest

The authors declare that the research was conducted in the absence of any commercial or financial relationships that could be construed as a potential conflict of interest.

## Publisher’s Note

All claims expressed in this article are solely those of the authors and do not necessarily represent those of their affiliated organizations, or those of the publisher, the editors and the reviewers. Any product that may be evaluated in this article, or claim that may be made by its manufacturer, is not guaranteed or endorsed by the publisher.

## Footnotes

1
https://github.com/statgen/bamUtil
